# In Vitro Studies of Pegylated Magnetite Nanoparticles in a Cellular Model of Viral Oncogenesis: Initial Studies to Evaluate Their Potential as a Future Theranostic Tool

**DOI:** 10.3390/pharmaceutics15020488

**Published:** 2023-02-01

**Authors:** Gabriel Principe, Virginia Lezcano, Silvina Tiburzi, Alicia B. Miravalles, Paula S. Rivero, María G. Montiel Schneider, Verónica Lassalle, Verónica González-Pardo

**Affiliations:** 1 Departamento de Biología, Bioquímica y Farmacia, Universidad Nacional del Sur (UNS), San Juan 670, Bahía Blanca 8000, Argentina; 2 Instituto de Ciencias Biológicas y Biomédicas del Sur (INBIOSUR), UNS-Consejo Nacional de Investigaciones Científicas y Técnicas (CONICET), Bahía Blanca 8000, Argentina; 3 Departamento de Química, Universidad Nacional del Sur (UNS), Avda. Alem 1253, Bahía Blanca 8000, Argentina; 4 Instituto de Química del Sur (INQUISUR), UNS-Consejo Nacional de Investigaciones Científicas y Técnicas (CONICET), Bahía Blanca 8000, Argentina

**Keywords:** viral cancer, nanoplatforms, magnetic nanoparticles, drug carrier, cytotoxicity

## Abstract

Magnetic nanosystems represent promising alternatives to the traditional diagnostic and treatment procedures available for different pathologies. In this work, a series of biological tests are proposed, aiming to validate a magnetic nanoplatform for Kaposi’s sarcoma treatment. The selected nanosystems were polyethylene glycol-coated iron oxide nanoparticles (MAG.PEG), which were prepared by the hydrothermal method. Physicochemical characterization was performed to verify their suitable physicochemical properties to be administered in vivo. Exhaustive biological assays were conducted, aiming to validate this platform in a specific biomedical field related to viral oncogenesis diseases. As a first step, the MAG.PEG cytotoxicity was evaluated in a cellular model of Kaposi’s sarcoma. By phase contrast microscopy, it was found that cell morphology remained unchanged regardless of the nanoparticles’ concentration (1–150 µg mL^−1^). The results, arising from the crystal violet technique, revealed that the proliferation was also unaffected. In addition, cell viability analysis by MTS and neutral red assays revealed a significant increase in metabolic and lysosomal activity at high concentrations of MAG.PEG (100–150 µg mL^−1^). Moreover, an increase in ROS levels was observed at the highest concentration of MAG.PEG. Second, the iron quantification assays performed by Prussian blue staining showed that MAG.PEG cellular accumulation is dose dependent. Furthermore, the presence of vesicles containing MAG.PEG inside the cells was confirmed by TEM. Finally, the MAG.PEG steering was achieved using a static magnetic field generated by a moderate power magnet. In conclusion, MAG.PEG at a moderate concentration would be a suitable drug carrier for Kaposi’s sarcoma treatment, avoiding adverse effects on normal tissues. The data included in this contribution appear as the first stage in proposing this platform as a suitable future theranostic to improve Kaposi’s sarcoma therapy.

## 1. Introduction

The transformation of a normal cell into a neoplastic one displays a set of characteristics that make them capable of aberrant proliferation, surviving by avoiding programmed cell death [1]. Viral neoplasms account for 12% of all human cancers and seven human oncoviruses have been recognized so far [2]. Oncoviruses carry viral oncogenes that regulate the growth and apoptosis of the host cells, as occurs in the development of non-viral cancers initiating the process towards complete transformation [3,4]. Neoplasms caused by the human herpesvirus-8 (HHV-8) or Kaposi’s sarcoma-associated herpesvirus (KSHV) are characterized by angiogenesis and the proliferation of spindle cells, which have qualities of activated endothelial cells [5,6,7,8]. Within the viral genome, the viral G protein-coupled receptor (vGPCR) drives oncogenesis and angiogenesis martin [5,8,9,10,11]; its persistent expression is necessary for tumor transformation and maintenance [9]. Although the incidence of AIDS-associated Kaposi’s sarcoma has decreased markedly since the widespread implementation of highly active antiretroviral therapy (HAART), a significant percentage of patients with this condition never achieve full remission [6,12,13]. Therefore, the vGPCR and its signaling pathways have become therapeutic targets both for the treatment of the pathology and for the development of new drugs. One of the problems of conventional cancer therapeutics and the application of new drugs is that they cannot differentiate between healthy and cancer cells, leading to systemic toxicity and undesired side effects.

Nanotechnology, in particular, provides different kinds of platforms aiming to offer more efficient and biosafe solutions to old pathologies. One of them concerns nanocarriers for several biopharmaceutical agents oriented to diverse pathologies, being the oncological ones preferred due to their well-known side effects and low efficacy [14,15,16]. Coating nanocarriers with targeted agents is an effective means of facilitating cell-specific internalization through binding between cancer cells and nanocarriers [17]. Magnetic drug targeting represents an attractive strategy that can be used to improve therapeutic efficacy in tumor cells, reducing not only side effects in normal cells and tissues, but also the dose required. Nanotheranostics represent promising alternatives to the traditional treatment procedures available for different pathologies. In this sense, magnetic nanoparticles (MNPs) based on iron oxides are highly suitable for the design of theranostics with improved properties such as magnetism (or superparamagnetism), biodegradability, relatively low toxicity, and ability to generate contrast in diagnostic imaging [18]. Moreover, the magnetic targeting of MNPs provides the possibility of directing therapeutic agents to the tumor area by the application of an external magnetic field [19]. From a therapeutic perspective, these magnetic nanodevices can induce the tumor cells’ death by magnetic hyperthermia. [18]. Novel approaches to improve the effectiveness of combined therapies have recently been published [20,21,22,23]. Several multifunctional nanoparticle systems have been synthesized and reported for cancer treatment [24,25]. Polyethylene glycol (PEG) has been recognized as an efficient coating for MNPs, especially those destined for biomedical applications. This polymer exhibits great biocompatibility and its effect on the retardation of nanoparticle elimination has been well reported [26].

In this work, we analyzed the biological action of pegylated iron oxide nanoparticles (MAG.PEG) to generate a non-toxic and efficient nanoplatform able to be loaded with different therapeutic agents for Kaposi’s sarcoma treatment. Although the information on the design and biomedical application of PEG coated magnetic nanoparticles has been sufficiently reported during the last years, the analysis of their performance in a cellular model of Kaposi’s sarcoma has not been deeply studied, according to the authors’ knowledge.

Physicochemical properties demonstrated that the MAG.PEG platform exhibited features suitable for biomedical application. According to in vitro assays, no alterations in cell proliferation and viability were observed at lower concentrations of MNPs (1–50 µg mL^−1^). Furthermore, MAG.PEG internalization occurs through an endocytic mechanism that retains the iron content in vesicles without inducing an increase in reactive oxide species in cytosol or cell death, at least at a concentration of 50 µg mL^−1^. In addition, targeting MAG.PEG to the tumor zone with a magnet would avoid adverse effects on normal tissues. The set of results arising from this work appears relevant to predicting the potential viability of MAG.PEG nanoplatform as a future nanotheranostic for Kaposi’s sarcoma treatment.

## 2. Materials and Methods

### 2.1. Chemicals and Reagents

The antibiotic G418 and the culture medium Dulbecco′s Modified Eagle′s Medium (DMEM) were from Sigma-Aldrich (St. Louis, MO, USA). The CellTiter 96^®^Aqueous One Solution Cell Proliferation Assay kit was from Promega (Tecnolab, Buenos Aires, Argentina). Ferric chloride hexahydrate, sodium dodecyl sulfate (SDS), and hematoxylin and eosin (H&E) dyes were provided by Biopack (Buenos Aires, Argentina). Ferrous sulfate heptahydrate and potassium ferrocyanide were from Mallinckrodt Chemical Works (Saint Louis, MO, USA). Sodium hydroxide and acetic acid were purchased from Cicarelli (San Lorenzo, Santa Fe, Argentina). Neutral red (3-amino-7-dimethylamino-2-methyl-phenazine hydrochloride), 2′,7′-dichlorofluorescin diacetate (DCFDA), osmium tetroxide, uranyl acetate, lead citrate, and Spurr’s resin were acquired by Sigma (Sigma-Aldrich, Saint Louis, MO, USA). All other reagents were of analytical grade.

### 2.2. Synthesis of Magnetic Nanoparticles

Polyethylene glycol 6000 solution at 2% *w*/*w* in distilled water was prepared. Then, iron compounds were added to this solution. The reaction mixture was magnetically stirred for 10 min at room temperature before the addition of NaOH 5M. Once the pH of the solution became higher than 10, the reaction mixture was placed into a Teflon-lined autoclave and heated in an oven at 160 °C for 9 h. After cooling to room temperature, nanoparticles were decanted over a neodymium magnet and washed with distilled water until the pH and conductivity reached values close to those of the distilled water [27].

A JEOL 100CX-II transmission electron microscope (JEOL, Akishima, Tokyo, Japan) was used to determine the particle size and morphology of the nanoparticles. Samples were dissolved in water, placed on 200 mesh Cu grids, and dried to room temperature. Data on hydrodynamic diameter and zeta (ζ) potential were acquired in a Malvern Zetasizer (Nano-Zs90). Samples were dispersed in distilled water and sonicated before the acquisition was performed, and before the biological assays.

### 2.3. Cell Lines and Culture Conditions

The experimental model of Kaposi’s sarcoma is represented by the SV-40-immortalized murine endothelial cells (SVEC cells) stably expressing the vGPCR full length receptor (vGPCR cells), as previously described [28]. This virally encoded receptor was found to promote tumor formation in immune-suppressed mice and to induce angiogenic lesions similar to those that occur in the development of Kaposi’s sarcoma [28,29]. vGPCR stably transfected cells were cultured in DMEM supplemented with 5% fetal bovine serum (FBS), 4.5 g L^−1^ glucose, and selected with 500 μg mL^−1^ G418. SVEC cells were cultured in DMEM with 10% FBS and 4.5 g L^−1^ glucose. Primary cultures of endothelial cells (EC) were obtained from mice aorta and gently donated by Dr. Pablo Cutini (Lab. Investigaciones Endócrinas Básicas y Clínicas, INBIOSUR, CONICET-UNS) and cultured in DMEM with 10% FBS and 1 g L^−1^ glucose. In all cases, the cells were allowed to grow in a metabolic incubator at 37 °C and 5% CO_2_ and the culture medium was replaced every two days.

### 2.4. Cell Proliferation Assays

#### 2.4.1. Crystal Violet Stain

The cells cultured in 48-well plates were incubated with different concentrations of MNPs (1–150 µg mL^−1^) or distilled water as control for 48 h. Then, the cells were washed with phosphate buffered saline or PBS (8 g L^−1^ NaCl; 0.2 g L^−1^ KCl; 1.44 g L^−1^ Na_2_HPO_4_; 0.24 g L^−1^ KH_2_PO_4_; pH 7.4), fixed with 4% paraformaldehyde for 5–10 min, and stained with 0.1% of crystal violet dye for 10 min at room temperature. Subsequently, the dye was quantified by adding Triton x-100 0.2% in water and the absorbance was measured at 590 nm in a microplate reader (Bioteck Synergy-HT).

#### 2.4.2. Trypan Blue Exclusion Assay

The vGPCR cells cultured in 24-well plates were incubated with different concentrations of MNPs (1–150 µg mL^−1^) or distilled water as control for 48 h. Then, non-adherent and attached cells were harvested as described by Lezcano and col. [30]. Briefly, the cells were then stained with 0.04% trypan blue and counted under microscope using a hemocytometer. Alive cells appeared unstained and colored cells indicated cellular death [31].

### 2.5. Cell Viability Assays

#### 2.5.1. Neutral Red Assay

The number of viable cells was quantitatively estimated by neutral red uptake assay following Repetto and col. protocol [32]. Briefly, vGPCR cells seeded in 96-well plates were treated with MNPs (1–150 µg mL^−1^) for 48 h. Then, the cells were incubated with the neutral red solution in DMEM for 3 h. Afterwards, micrographs of each condition were acquired under an inverted light field microscope (Nikon TE300 Eclipse) equipped with a digital camera. Then, the absorbance at 540 nm was measured in a microplate reader (Bioteck Synergy-HT).

#### 2.5.2. MTS Assay

The vGPCR cells were seeded in 96-well plates and incubated with different concentrations of MNPs (1–150 µg mL^−1^) for 48 h. Then, the medium was removed and 120 µL of the tetrazolium inner salt, MTS (CellTiter 96^®^ AQueous one solution cell proliferation assay) prepared in 2% FBS medium was added per well according to the manufacturer’s instructions. The absorbance was measured at 490 nm in a microplate reader (Bioteck Synergy-HT).

### 2.6. Iron Detection and Quantification by Prussian Blue Assay

The vGPCR cells were cultured in 48-well plates and incubated with MAG.PEG (1–150 µg mL^−1^) for different periods of time (3–48 h). Then, the cells were washed with PBS and fixed with 4% paraformaldehyde for 10 min at room temperature. Subsequently, the cells were washed and incubated with the Prussian blue solution (5% K_4_Fe (CN)_6_; 1% HCl) for 15 min and counterstained with H&E. Micrographs from each concentration were obtained by an inverted light field microscope (Nikon TE300 Eclipse) equipped with a digital camera. In parallel experiments, the iron content of MAG.PEG was quantified by the incubation of the vGPCR cells with 250 µL of 20% HCl overnight at 37 °C. Next, 250 µL of 10% K_4_Fe (CN)_6_ was added per well and the absorbance was measured at 700 nm in a microplate reader (Bioteck Synergy-HT).

### 2.7. Quantification of Reactive Oxygen Species (ROS)

The cellular oxidant levels were determined by using the fluorogenic dye 2′,7′-dichlorofluorescin diacetate (DCFDA) that measures hydroxyl, peroxyl, and other ROS products within the cell. Briefly, this dye, when crossing the membrane, is deacetylated by cellular esterases and then oxidation by ROS to convert it into a fluorescent compound [33]. The vGPCR cells were cultured in 48-well plates and incubated with MAG.PEG (10, 50 and 150 µg mL^−1^) for 24 h. In addition, 0.5 mM H_2_O_2_ treated cells for 45 min as positive control was employed. Then, the medium was replaced with fresh medium containing 10 µM of DCFDA for 30 min at 37 °C. Next, the cells were rinsed three times with free FBS medium and thereafter the fluorescence was measured in a microplate reader (λ Ex/Em= 485(20)/528(20) nm, Bioteck Synergy-HT). Afterwards, the cellular protein content was obtained by lysing the cells with a PBS buffer containing 1% Nonidet P-40 and quantified by the Bradford method [34]. Finally, the fluorescence intensity was normalized by the corresponding total protein content.

### 2.8. Transmission Electron Microscopy (TEM): Sample Preparation

The vGPCR cells were cultured in a 12-well plate and incubated with MAG.PEG (50 µg mL^−1^) at different periods of time (1–48 h), washed with PBS, and fixed with 1% glutaraldehyde and 3% paraformaldehyde in PBS for 1.5 h at 5 °C. Fixation was followed by a series of rinses with PBS; then, a post-fixation was performed with 1% osmium tetroxide in PBS for 40 min and finally washed with PBS. Thereafter, the cellular monolayer was scrapped and incubated with 1% aqueous uranyl acetate for 40 min, washed again, and dehydrated with a gradually increasing series of acetone (30–100%). Finally, the cells were infiltrated in Spurr’s resin over 4 days and polymerized for 12 h at 70 °C. Ultrathin sections were obtained with an Ultracut Reichert Jung ultramicrotome and stained with aqueous uranyl acetate followed by lead citrate. Sections were observed in a JEOL 100CX-II transmission electron microscope (JEOL, Akishima, Tokyo, Japan) operated at 80 kV at the CCT-CONICET Bahía Blanca (Argentina).

### 2.9. Application of a Static Magnetic Field (SMF)

The vGPCR cells were cultured in a 35 mm petri dish and incubated with 10 or 150 µg mL^−1^ of MAG.PEG or distilled water as control in the presence or absence of a SMF for 48h. The SMF was generated by a 5 mm diameter NdFeB magnet (0.3 Tesla) located under the petri dish. Afterwards, the magnet was removed. The cells were washed and fixed with 4% paraformaldehyde. To evidence the MNPs, the Prussian blue technique was performed as described in Section 2.6 and then the cells were counterstained with H&E [15]. Micrographs were captured using an inverted light field microscope (Nikon TE300 Eclipse) equipped with a digital camera.

### 2.10. Statistical Analysis

The statistical analysis was performed using R Studio. Shapiro–Wilk and Levene tests were used to evaluate normality and homoscedasticity, respectively. When applicable, one-way ANOVA and Dunnett post hoc tests were performed to determine the statistical significance between MNPs conditions and the control group. In addition, to compare the mean between two conditions, Student’s *t*-test was used. When the data was not normally distributed, the Kruskal–Wallis test was performed, followed by the Mann–Whitney U test. To analyze count data, the Chi-square test was used. The data wetr represented as mean ± SD in bar graphs; or in a box plot. The statistical significance is shown as * *p* < 0.05; ** *p* < 0.01; *** *p* < 0.001 vs. control.

## 3. Results

### 3.1. Chemical Characterization of MAG.PEG Nanosystem

**Figure 1:**
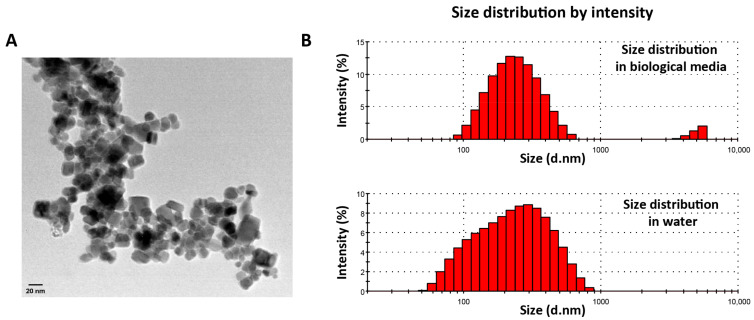
**TEM micrographs of MAG.PEG**. (**A**) A representative micrograph of the MAG.PEG dispersion is shown. The morphology and size of nanoparticles is appreciated with this technique. Scale bar = 20 nm. (**B**) Histograms arising from DLS analysis.

The size of the magnetic nanoparticles is critical for their application in biomedicine. Magnetic nanoparticles usually present two parts: the magnetic core, composed in this case of magnetite/maghemite iron oxide; and the shell, which usually has multiple functions as biocompatibilizer, functionalizer, and/or stabilizer [16]. One of the widely used techniques to measure the size of the core is transmission electron microscopy (TEM). The size of the inorganic core is related to the magnetic properties of the nanoparticles since nanoparticles between 25–40 nm exhibit superparamagnetism [35]. TEM also allows us to observe the morphology of the nanoparticles. Figure 1A shows the micrograph corresponding to MAG.PEG. It is observed that the nanoparticles, in general, present a more or less cubic morphology with a determined size of 23.5 nm.

The zeta potential of MAG.PEG was −22.1 mV measured at pH 5.5 in distilled water. This value is the expected one based on the amphoteric nature of MAG and the steric stabilization induced by the PEG chains.

For biological applications, it is important to determine the hydrodynamic diameter, which is the size that the nanoparticles acquired in aqueous dispersions. For this purpose, it is generally accepted that nanoparticles with a hydrodynamic diameter between 10 and 300 nm are adequate for their use in biomedicine [36]. MAG.PEG shows a hydrodynamic diameter of 208 nm measured in distilled water, while the size in biological media—Dulbecco′s Modified Eagle′s Medium supplemented with 2% of fetal bovine serum (DMEM with 2% FBS)—was 236.6 nm. The histograms arising from DLS analysis, and included in Figure 1B, demonstrated that the samples are roughly monodisperse in these media, registering PDI values of 0.295 and 0.315 for MAG and MAG.PEG in biological media, respectively. These data demonstrate that almost monodisperse nanosystems were obtained. It is important to highlight that the exhaustive physicochemical characterization of these nanosystems has been included in a separate work; in the present contribution, the focus is occupied by the biological study of their performance faced with an innovative nanodevice for Kaposi’s sarcoma.

### 3.2. Biological Studies to Evaluate the MNPs Cytotoxicity in vGPCR Cells

**Figure 2:**
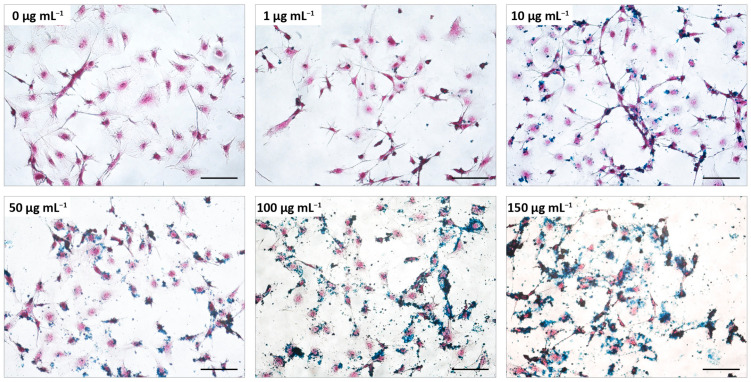
**Effect of MAG.PEG on the cell morphology**. The vGPCR cells were incubated with different concentrations of MAG.PEG (1–150 μg mL^−1^) or distilled water as control for 48 h. The cells were stained with H&E and the MNPs were evidenced by the Prussian blue reaction. Representative micrographs from each concentration were obtained by an inverted light field microscope. Scale bar = 100 µm. Magnification 200×.

As a first step to characterize the biological effects of the MNPs on the cellular model of Kaposi’s sarcoma, the morphology of the cells was analyzed. For this, the vGPCR cells were incubated with different concentrations of MAG.PEG (1–150 µg mL^−1^) or distilled water as control for 48 h. Next, the cells were stained with H&E to visualize the cytoplasm and the nuclei and with Prussian blue to evidence the iron of the MNPs. The cell morphology was then observed by light field microscopy and micrographs were taken with a digital camera. As can be seen from Figure 2, the vGPCR cells displayed the typical spindle shape and size and these characteristics were preserved in the presence of the MNPs regardless of the concentration employed. Furthermore, an increment in the blue precipitation that directly correlates with the MNPs concentration can be observed over and surrounding the cells.

**Figure 3:**
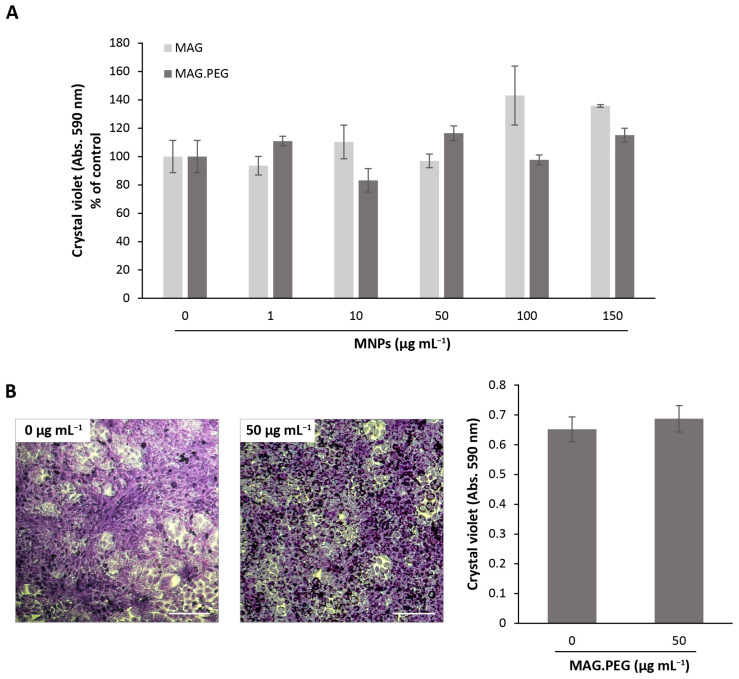
**Cell proliferation analysis**. The vGPCR cells were incubated with different concentrations of MNPs (1–150 μg mL^−1^) or distilled water as control for 48 h (**A**) or in the presence/absence of MAG.PEG (50 μg mL^−1^) for 120 h (**B**). To determine the proliferation, the cells were stained with crystal violet and thereafter the colorant was quantified at 590 nm. (**A**) The data is presented as the percentage of control ± SD. Non-statistically significant differences were assessed by Kruskal–Wallis test. N = 3. (**B**) Representative micrographs were obtained by an inverted light field microscope. Scale bar = 50 µm. Magnification 100×. Absorbance data is presented as mean ± SD. Non-statistically significant differences were identified by Student’s *t*-test. N = 4.

The next study was to determine whether the MNPs provoke changes in the cell proliferation and to analyze cell toxicity. Thus, the vGPCR cells were incubated with different concentrations of MNPs (1–150 µg mL^−1^) with (MAG.PEG) or without coating (MAG) for 48 h. The proliferation was then evaluated indirectly by crystal violet assay. As shown in Figure 3A, the quantification of the dye revealed that the MNPs did not induce statistically significant changes in the vGPCR cells’ proliferation regardless of the coating or the concentration used. Furthermore, this finding was also corroborated by the trypan blue exclusion assay (Figure S1). Additionally, proliferation experiments performed under the same conditions in primary cultures of endothelial cells (EC) and in SVEC cells, which do not express vGPCR, unearthed the same conclusions (Figure S2). To follow up whether the proliferation remained unchanged for a longer period of time, the vGPCR cells were incubated with 50 µg mL^−1^ of MAG.PEG for 5 days and then stained with crystal violet dye. As expected, comparing Figure 2 and Figure 3B, the number of cells increased due to the time of culture and no differences between the control and MAG.PEG conditions were observed. This finding was supported by the quantification of the crystal violet dye, indicating that the presence of the MNPs did not modify the cellular proliferation. In addition, representative micrographs shown in Figure 3B reveal that the cell morphology was unaffected in the presence of MNPs compared to the control. What is more, MNPs aggregates were evidenced in the perinuclear zone.

**Figure 4:**
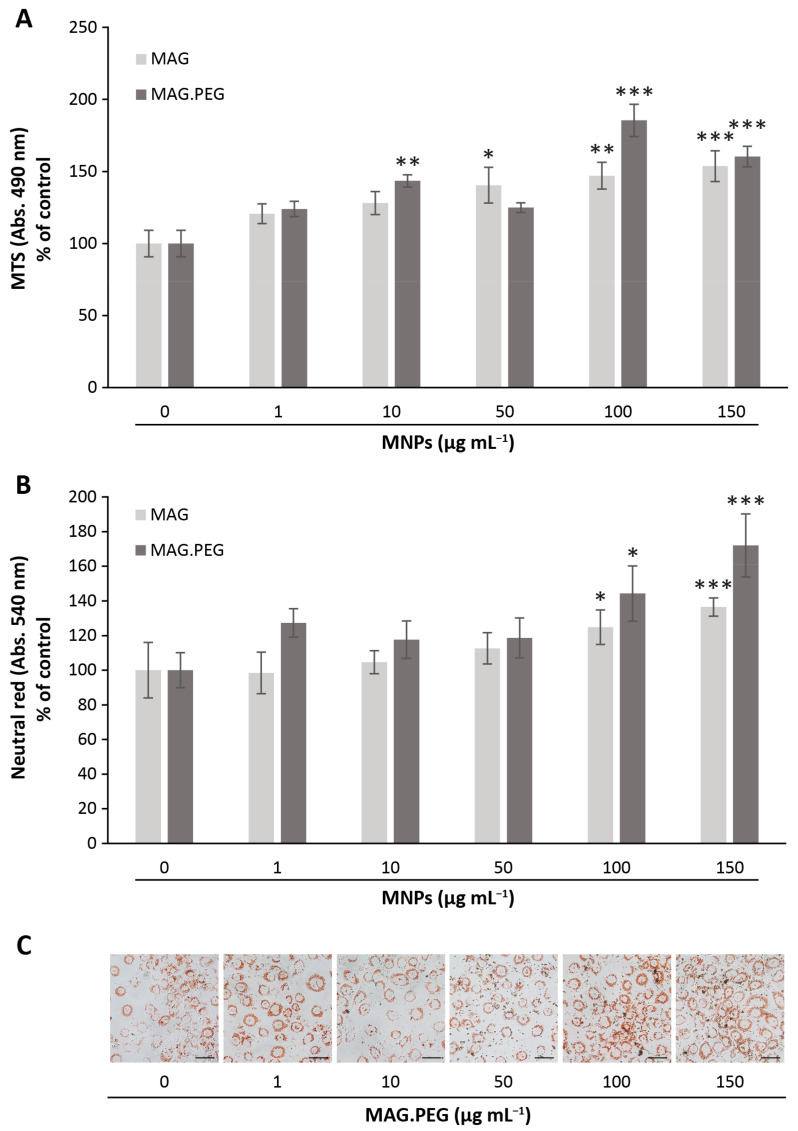
**Cell viability assays**. The vGPCR cells were incubated with different concentrations of MNPs (1–150 μg mL^−1^) or distilled water as control for 48 h. (**A**) The metabolic activity of the cells was measured by MTS as described in the Methods section. Absorbance was measured at 490 nm. The data is presented as the percentage of control ± SD. N = 3. (**B**) Lysosomal activity of the cells was measured by neutral red assay as described in the Methods section. Absorbance was measured at 540 nm. The data is presented as the percentage of control ± SD. N = 5. (**A**,**B**) Significant differences between control and MNPs conditions were evaluated by ANOVA and Dunnett post hoc tests, and are indicated as * *p* < 0.05, ** *p* < 0.01, and *** *p* < 0.001. (**C**) Representative micrographs of the cells incubated with different concentrations of MAG.PEG and stained with neutral red were obtained by a phase contrast microscope. Scale bar = 50 µm. Magnification 200×.

Lastly, cell viability studies were conducted to analyze the metabolic activity of the vGPCR cells in the presence of MNPs. For this, the vGPCR cells were incubated with MAG.PEG or MAG at different concentrations (1–150 µg mL^−1^) or distilled water as control for 48 h; thereafter, the viability of the cells was measured by two complementary methods. Firstly, the dehydrogenase activity of active metabolic cells was determined by the reduction of MTS tetrazolium salt to a colored product (formazan). The results shown in Figure 4A indicate that the presence of both MAG and MAG.PEG prompted a statistically significant increase in the dehydrogenase reduction activity, being highly statistically significant at 150 µg mL^−1^ of MAG, and 100 and 150 µg mL^−1^ of MAG.PEG. Secondly, the capacity of viable cells to uptake the neutral red dye was studied. This method is based on the capacity of the dye to penetrate cell membranes being retained in the lysosomes of active cells [32]. As illustrated by Figure 4C, the neutral red was accumulated in lysosomal vesicles surrounding the nucleus of vGPCR cells incubated with MAG.PEG (1–150 µg mL^−1^). Moreover, an increase in the number of vesicles and color intensity was observed at high concentrations of the MNPs (100 and 150 µg mL^−1^). The neutral red was then solubilized and quantified at 540 nm (Figure 4B). The results indicate that a statistically significant increase of lysosomal activity was achieved by both, MAG and MAG.PEG, at 100 and 150 µg mL^−1^.

### 3.3. Evaluation of Reactive Oxygen Species (ROS) Production by MNPs

**Figure 5:**
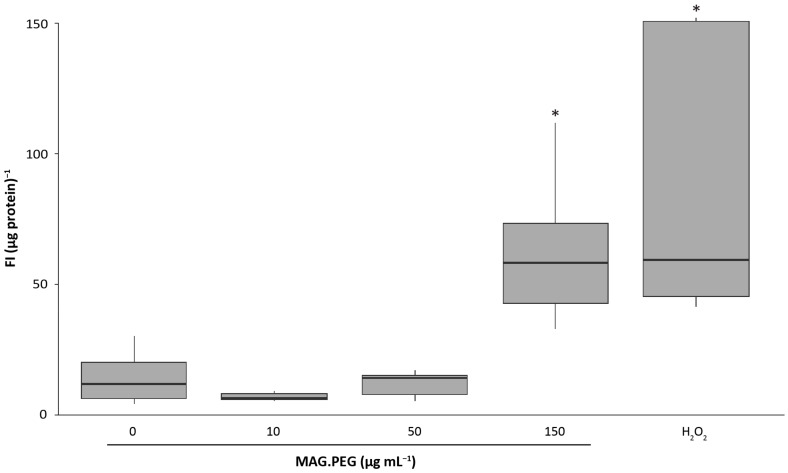
**Detection of ROS**. The vGPCR cells were cultured in 48-well plates and incubated with MAG.PEG (10, 50 and 150 µg mL^−1^) for 24 h or 0.5 mM H_2_O_2_ for 45 min as positive control. Then, 10 µM of DCFDA was added to the medium as described in the Methods section and then the fluorescence was measured in a microplate reader. The fluorescence intensity (FI) of each condition was normalized by the total protein content and represented in a box plot. Kruskal–Wallis followed by the Mann–Whitney U test was performed, and the statistically significant differences between control and MAG.PEG conditions are indicated as * *p* < 0.05. N = 6.

ROS levels have become relevant in the oncogenesis process. It has been reported that endothelial cells expressing vGPCR have high levels of ROS, which are important to stimulate angiogenesis [9]; however, a high concentration of ROS is cytotoxic in healthy cells. Since it has been shown that MNPs induce ROS production and consequently cytotoxicity [37], we investigated whether the range of MNPs concentration employed in this work increases the ROS levels in vGPCR cells. Thus, the cells were incubated with MAG.PEG (10, 50 and 150 µg mL^−1^) or distilled water as control for 24 h. From Figure 5, it can be seen that only high concentrations of MAG.PEG (150 µg mL^−1^) induced a significant augment in ROS levels compared to the control. Similar results were obtained when vGPCR cells were treated with 0.5 mM of H_2_O_2_ for 45 min, employed as positive control.

### 3.4. Analysis of the Cellular MNPs Uptake and Localization

**Figure 6:**
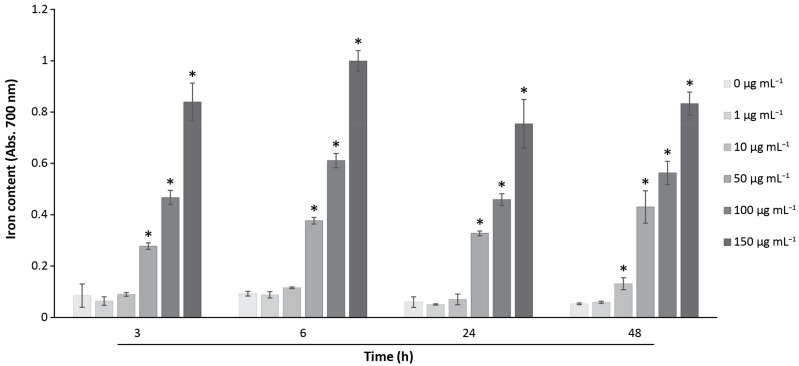
**Iron quantification of MAG.PEG**. The vGPCR cells were incubated with different concentrations of MAG.PEG (1–150 μg mL^−1^) or distilled water as control in settled periods of time (3–48 h). The iron content was determined by Prussian blue assay and the colored product was measured at 700 nm. The data is presented as mean ± SD in a bar graph. Kruskal–Wallis followed by the Mann–Whitney U test was performed, and the statistically significant differences between control and MAG.PEG conditions are indicated as: * *p* < 0.05. N = 4.

From the qualitative analysis of the iron content of MNPs shown above in Figure 2, further quantitative studies were conducted to analyze the kinetic profile of the iron content at different concentrations. The vGPCR cells were incubated with different concentrations of MAG.PEG (1–150 μg mL^−1^) for different periods of time (3–48 h). Then, the cells were stained with Prussian blue dye and the colorant was quantitatively measured as described in the Methods section. The results presented in Figure 6 show that the iron content increased as the concentrations of MAG.PEG were progressively augmented at all the points of time studied. This effect became statistically significant in a concentration range of 50–150 μg mL^−1^ of MAG.PEG with respect to the control. Moreover, to achieve a statistically significant effect with a lower concentration (10 μg mL^−1^) of MNPs, a longer incubation time was necessary (48 h).

**Figure 7:**
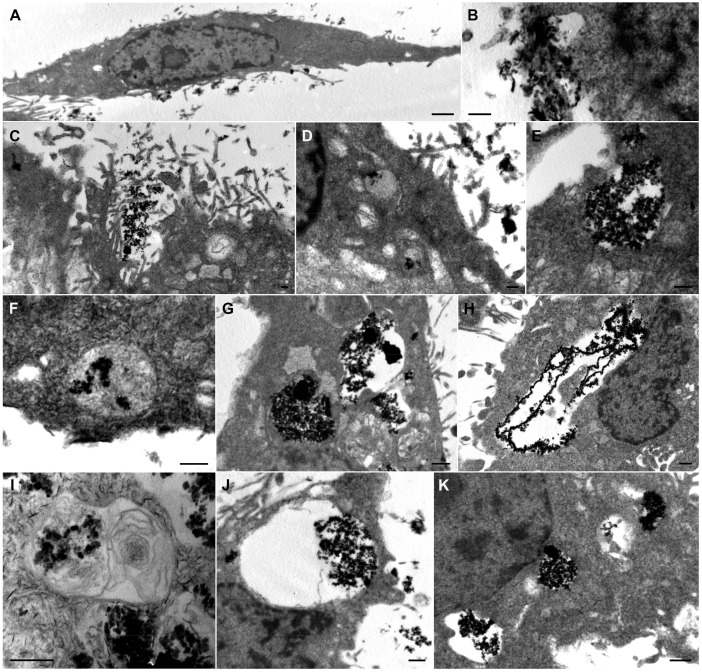
**MAG.PEG uptake and cellular distribution by TEM**. The vGPCR cells were incubated with MAG.PEG for 1 h (**B**), 24 h (**A**,**C**–**I**), and 48 h (**J**,**K**). (**A**) Electron micrograph of an endothelial cell displaying the typical morphology. (**B**,**C**) MNPs aggregates outside the cells and near the plasma membrane. (**D**) Small sized vesicles with few particles inside. (**E**–**K**) Electron micrographs of vesicles of different size and shape containing MNPs aggregates. (**E**) Translucent vesicle near the membrane. (**F**) Relatively dense vesicle near the membrane. (**G**) Both types of vesicles with large amounts of particles. (**H**) Clusters of particles near the nucleus. (**I**) Vesicles with multilamellar membranes. (**J**) Large vesicle with aggregates of particles close to the nucleus. (**K**) Several vesicles with aggregates close to the nucleus. Scale bars: (**A**) = 1 μm; (**B**–**F**,**I**) = 0.2 μm; (**G**,**H**,**J**,**K**) = 0.5 μm.

To decipher MNPs localization and distribution within the cells, TEM was employed. Thus, the vGPCR cells were incubated with 50 μg mL^−1^ of MAG.PEG or control for different points of time (1, 24 and 48 h). Figure 7A shows a typical spindle endothelial cell. The MNPs can be seen aggregated outside the cell very close to the plasma membrane (Figure 7B,C) after short periods of incubation time. Small amounts of particles inside small endocytic vesicles can be seen within the cell close to the membrane (Figure 7D). Moreover, endocytic vesicles and vesicles with different sizes and quantities of MNPs can be observed after longer periods of incubation time (Figure 7E–J). The vesicles containing clusters of MNPs show different morphologies, from translucent (Figure 7H,J) towards much denser vesicles (Figure 7G) and even multi-lamellar ones (Figure 7I). Moreover, the number of vesicles with larger MAG.PEG aggregates were accumulated close to the nuclei (Figure 7H,J,K). What is more, no free cytoplasmic MNPs were identified.

### 3.5. Steering of MNPs by the Application of a Static Magnetic Field

**Figure 8:**
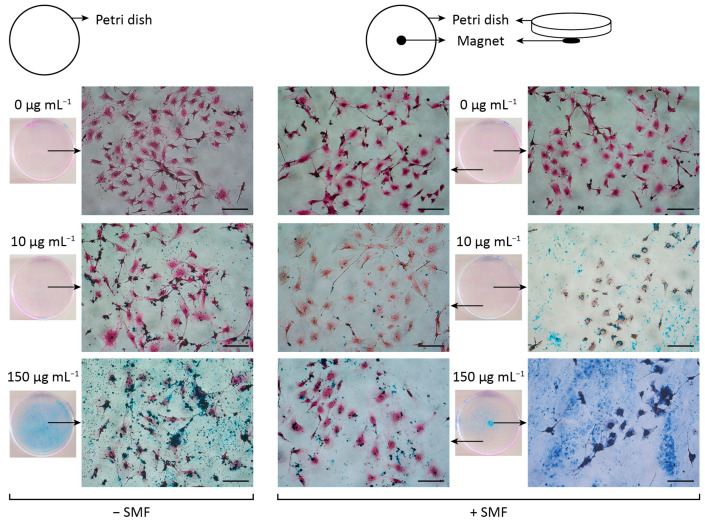
**MAG.PEG steering by the application of an external static magnetic field (SMF)**. The vGPCR cells were incubated with 10 or 150 µg mL^−1^ of MAG.PEG or distilled water as control in the presence (right panel) or absence (left panel) of a SMF for 48 h. Representative micrographs of the cells after the Prussian blue and H&E stain were taken under an inverted light field microscope. Arrows displayed from the culture dishes indicate the area of the micrograph acquisition. Scale bar= 100 µm. Magnification 200×.

One of the principles of magnetic drug targeting is to carry the drug loaded in MNPs and use an external static magnetic field (SMF) to guide the drug to the desired location [38]. Thus, the steering of MAG.PEG with a low and a high concentration of MNPs was analyzed in vGPCR cell cultures. The cells were incubated with 10 or 150 µg mL^−1^ of MAG.PEG or distilled water as control for 48 h in the presence or absence of a SMF generated by a magnet, as described in the Methods section. As shown in Figure 8, the guidance of MNPs to the center of the petri dish was achieved by the presence of the magnet and this effect was more evident with the highest concentration employed. From the analysis of the micrographs, it can be observed that in the absence of the SMF, the MNPs remained dispersed all over the petri dish. In contrast, the arrangement of the MNPs was different in the presence of the SMF; this behavior was evidenced by the accumulation of MNPs in the cells grown over the magnet resulting in its decrement in the cells grown distant from the magnet.

## 4. Discussion

As was mentioned in the introduction, current treatments for the malignancy caused by an oncovirus such as Kaposi’s sarcoma-associated herpesvirus (KSHV) are not completely efficient. Little is known in the literature about the uses of nanosystems for therapeutic purposes in these types of oncogenic cancers. In this regard, silver nanoparticles have been demonstrated to cause cytotoxicity in KSHV infected cells, reactivating viral lytic replication and provoking the induction of ROS generation and autophagy [39]. Furthermore, only one report compared the effects of pegylated liposomes loaded with doxorubicin and liposomal daunorubicin on the treatment of advanced-stage Kaposi’s sarcoma; unfortunately, and because of the power of the study, there is no sufficient evidence of the efficacy of these treatments [40]. More recently, strategies to delivery chemotherapeutic drugs used for sarcoma treatment have been developed [41]. One strategy is focus on the development of paclitaxel nanocrystal formulation to improve the water solubility properties of this hydrophobic drug [42]. In another case, low-frequency ultrasound irradiation was used to improve the efficacy of paclitaxel to induce apoptosis of sarcoma cells [43]. Another strategy for solid tumor treatment has emerged using a novel extracellular matrix (ECM)-targeting nanotherapeutic. In this system, an inhibitor of the ECM is linked to lipid-based NP [44]. In this work, MNPs composed of magnetite have been chosen among other nanoformulations because of its appropriate characteristics related to size, magnetic behavior, and the ability to be internalized by cells and tissues [45]. One of the drawbacks of using magnetite alone for in vivo studies is the rapid elimination by renal clearance for NPs smaller than 5 nm or the absorption by the reticuloendothelial system of the liver and spleen for NPs ranging from 10 to 200 nm [46]. In addition, in vivo studies have well demonstrated that macrophages can remove the MNPs from the blood [47,48]. Thus, functionalization of MNPs with different coatings attempt to overcome this problem. Among them, the positive effects of coating nanoparticles with PEG have been widely described. Specifically, it is known that NPs coated with PEG acquire a hydrophilic shell that helps them to avoid rapid phagocytosis [47]. PEG also stabilizes the magnetic core and can ameliorate the absorption and release of active molecules from the MNPs [45].

Many reports indicate that several types of serum protein interact with circulating NPs forming a protein corona on their surface, which facilitate their uptake by cells (even also in endothelial cells) during their circulation [49,50]. In this regard, pegylation has shown to increase the hydrodynamic size because of the adsorption of plasma proteins. In this work, this parameter was measured as part of the characterization of our nanosystem; in agreement with other authors [51], the hydrodynamic size of MAG.PEG was higher when it was measured in biological media containing FBS compared to distilled water, meaning that the protein corona has been formed. In both cases, the size obtained was appropriate for biomedical applications. Based on the mentioned characteristics, pegylated MNPs are an adequate nanoplatform to approach with biological assays in order to validate their status as a potential drug nanocarrier for the treatment of Kaposi’s sarcoma.

One of the key points in the study of the biological effects of nanoparticles is to determine the appropriate range of concentrations and the incubation time in their use as a non-toxic carrier. These parameters may vary according to cell types. For example, in an in vitro study using human breast cancer cells (MCF-7) and adenocarcinoma lung cancer cells (A549 cells), it was determined that a concentration range of 40–370 µg mL^−1^ of MNPs functionalized with PEG has no cytotoxic effects, regardless the incubation time (24–48 h) [45]; in another study, also using MCF-7 cells, it was reported that MNPs coated with PEG began to be toxic at 500 µg mL^−1^ [52]. In this work, we found that the incubation of endothelial vGPCR cells with MNPs in a concentration range of 1–150 µg mL^−1^ does not alter the proliferation; however, at the higher concentrations (100–150 µg mL^−1^), an increase in cell viability was observed. This does not lead to an increase in proliferation, but rather correlates with an increase in the uptake of MNPs that generates a rise in metabolic and lysosomal activity. This observation is also supported by the results of the Trypan blue exclusion assay, which demonstrated that the proportion of living cells was not modified by the presence of MNPs. In line with our results, Hsieh and col. [53] reported that in mouse macrophage cells and MCF-7 cells, at 100 µg mL^−1^ of SPION-PEG, the mitochondrial activity was increased despite no cytotoxic effects being observed. In addition, the authors associated the increased mitochondrial activity with ROS generation at the same concentration due to the presence of SPIONs-PEG in this organelle which turned out in cell death by apoptosis. We found that in vGPCR cells, the ROS levels were augmented at 150 µg mL^−1^ of MAG.PEG, similar to the positive control. This finding could be correlated with the high metabolic and lysosomal activity found at the same concentration.

The incorporation of MAG.PEG into vGPCR cells was first observed by Prussian blue stain after 48 h, where MNPs appear distributed as spots of different sizes, increasing their accumulation as the concentration of MNPs augmented. Similar results have been reported for MNPs with different coatings; for example, in MCF-7 cells [54] and in human cervical adenocarcinoma cells [55,56]. In depth, our quantitative studies of the Prussian blue stain revealed that the accumulation of MAG.PEG occurs rapidly within 3 h and the iron content correlates with the MNPs concentration rather than the time of incubation. Furthermore, to obtain a significant increase with a lower concentration (10 µg mL^−1^), a longer period of time was required (48 h). There are many reports regarding the MNPs-labeling efficiency; nevertheless, it is difficult to compare them because of the different protocols, sizes, coatings of the MNPs, incubation times, cell lines, and other variables. In general, and in agreement with our findings, there is an association between incubation time and MNPs concentrations that leads to a higher intracellular accumulation of the MNPs within the cells [54,57,58]. Nevertheless, the exposure of cells to high concentrations of MNPs for prolonged times may cause cytotoxicity [54]. Therefore, it is important to achieve a sufficient intracellular uptake of MNPs in balance with their biocompatibility to be an efficient drug carrier [56].

In this regard, many types of in vitro and in vivo studies were conducted to explore the toxicity of the MNPs [59,60,61]. In general, MNPs have been shown to enhance ROS intracellular production which turned out in cell damage and death [62,63]. In this sense, as was mentioned before, our results indicated that with a high concentration of MAG.PEG (150 µg mL^−1^), an increase in ROS production was observed; thus, the incubation of the cells for prolonged periods of time may produce undesired cell death or damage. For internalization experiments using TEM, therefore, a concentration of 50 µg mL^−1^ of MAG.PEG was used. We confirmed the incorporation and accumulation of MNPs in endocytic vesicles without identifying free cytoplasmic MNPs, even after 48 h of incubation. This may explain the lack of increase in ROS production in the cells incubated with this concentration of MNPs.

NPs can accumulate into the tumor tissue through passive or active targeting. In the first case, the accumulation is possible due to enhanced permeability and retention effect. This phenomenon appears as a consequence of both the high permeability of the tumor vasculature and the ineffective lymphatic drainage. Conversely, active targeting can be accomplished by several ways, such as the introduction of a ligand that can be recognized by overexpressed receptors in tumor cells [64], and magnetic guidance achieved by the application of an external static magnetic field (SMF). The benefit of this active targeting is the reduction of side effects in healthy cells and tissues [19]. Most in vivo studies have demonstrated successful targeting of MNPs towards the tumor by the application of an SMF of a strength between 0.2 and 0.6 T, with different times of application ranging from 30 min [65] to 48 h [66]. In this work, we studied the effect of the presence of an SMF generated by a magnet of 0.3 T during the incubation of vGPCR cells with two concentrations of MAG.PEG for 48 h. Our results revealed that the pattern of distribution of the MNPs in the cell cultures exposed to the SMF is different from that observed in the absence of the magnet. Of interest, we found that the quantity of MNPs drastically decreases in areas far from the center of the cell culture, which would allow for the achievement of a controlled administration of drugs on the tumor, reducing the effect on neighboring healthy tissues. Supporting this idea, there is already in vivo evidence of targeting MNPs using an extracorporeal magnet with promising results; for example, in mice [67], rats [68], and humans [69]. Moreover, comparing not only the conditions with or without magnet, but also the different areas of the cell culture, it was observed that the cell morphology remained unchanged. Similar results were obtained by Martin and col. [15] in a different cellular model. However, Bae and col. [70] demonstrated a synergistic response after SPION and SMF of 0.4 T in normal mouse hepatocytes.

Despite the promising in vitro data emerging from this work, some limitations might arise in in vivo experiments; care should therefore be taken in extrapolating these results. In this sense, some variables should be considered, such us the accumulation of MNPs in healthy tissue regardless of the magnetic targeting, the reduced blood perfusion found in some type of sarcomas, and the difficulty of magnetic targeting due to tissue thickness, among others. Nevertheless, addressing these concerns exceed the scope of our work. 

## 5. Conclusions

Analyzing the results obtained in this study and comparing them with the findings of other authors, one of the evident conclusions is the heterogeneous behavior of MNPs in biological systems; hence, it is important to take into account the concentrations and incubation times in carrying out the biological tests desired, along with the cell type employed. In this work, we used a cellular model of Kaposi’s sarcoma to validate MAG.PEG as a nanoplatform. We found that in a range of 10–50 µg mL^−1^, MNPs did not increase ROS production and did not cause significant effects in lysosomal activity, suggesting that MNPs would be an efficient and biocompatible drug carrier in this range of concentration. Finally, the targeting of these MNPs to the tumor area would avoid adverse effects on healthy tissues.

## Data Availability

Not applicable.

## Supplementary Materials

The following are available online at https://www.mdpi.com/article/10.3390/pharmaceutics15020488/s1, Figure S1: Trypan blue exclusion assay for cell proliferation, Figure S2: Crystal violet assay for cell proliferation.

## Abbreviations

KS: Kaposi’s sarcoma; vGPCR, viral G protein-coupled receptor; MNPs, magnetic nanoparticles; MTS, (3-(4,5-dimethylthiazol-2-yl)-5-(3-carboxymethoxyphenyl)-2-(4-sulfophenyl)- 2H-tetrazolium inner salt); AIDS, acquired immunodeficiency syndrome; HIV, human immunodeficiency virus; SPION, superparamagnetic iron oxide nanoparticles; NPs, nanoparticles.
