# Supplementary figures and images for: In Vitro Studies of Pegylated Magnetite Nanoparticles in a Cellular Model of Viral Oncogenesis: Initial Studies to Evaluate Their Potential as a Future Theranostic Tool
Source: Pharmaceutics. 2023 Feb 1;15(2):488. doi: 10.3390/pharmaceutics15020488 (PMC9967771; doi:10.3390/pharmaceutics15020488)

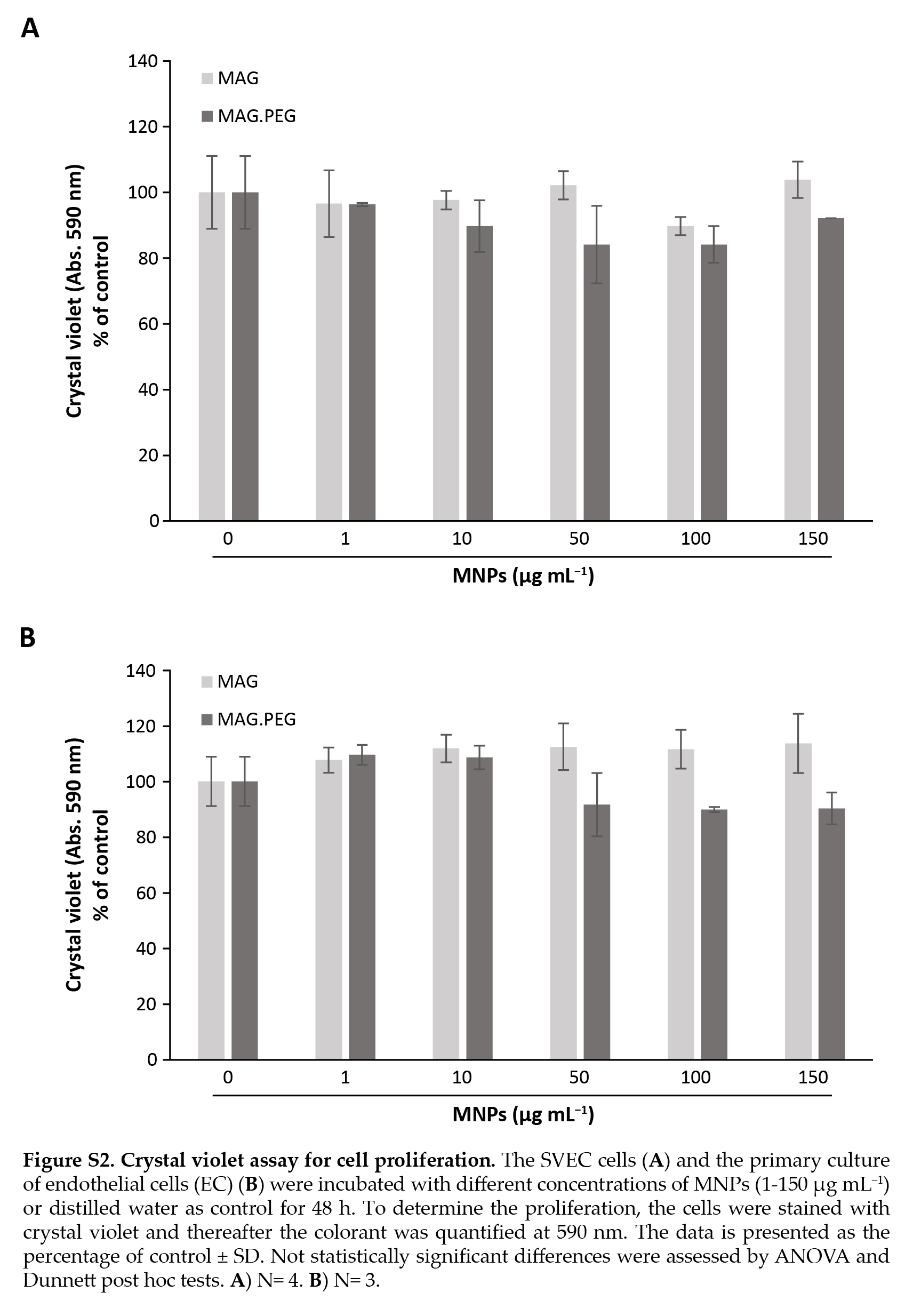

Supplement: Supplementary file 1 [file pharmaceutics-15-00488-s001.zip › Figure S2.tif]

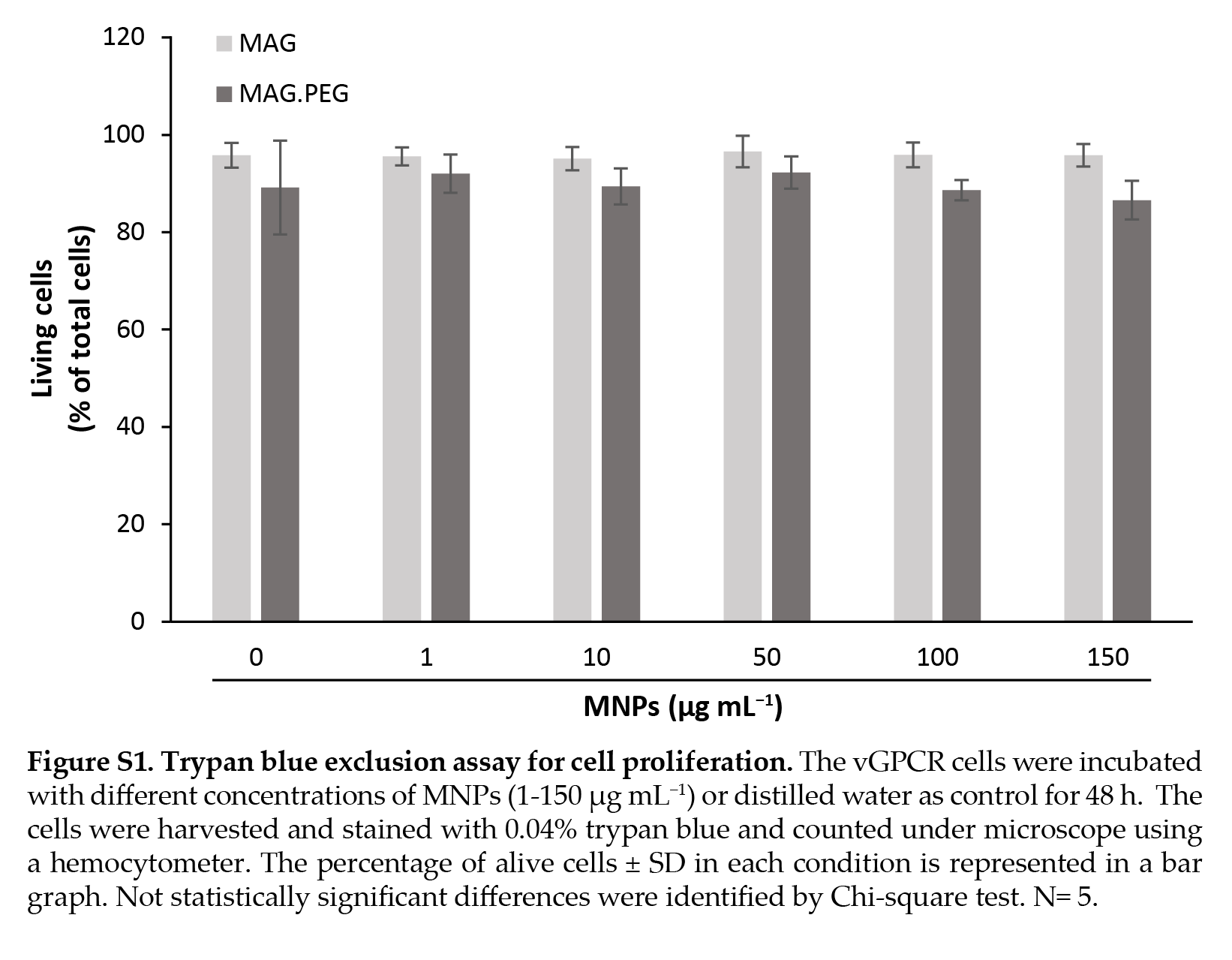

Supplement: Supplementary file 1 [file pharmaceutics-15-00488-s001.zip › Figure S1.tif]
